# Determinants of the Need for Tracheostomy in Neurocritical Patients

**DOI:** 10.7759/cureus.11654

**Published:** 2020-11-23

**Authors:** Isabel Taveira, Raul Neto, Pedro Salvador, Rita Costa, Paula Fernandes, Paula Castelões

**Affiliations:** 1 Internal Medicine, Hospital do Litoral Alentejano, Santiago do Cacém, PRT; 2 Internal Medicine, Centro Hospitalar Vila Nova de Gaia, Vila Nova de Gaia, PRT

**Keywords:** tracheostomy, mechanical ventilation, ventilator weaning, critical care

## Abstract

Background: Given the difficulties in predicting the need for prolonged intubation and the timing of tracheostomy, the stroke-related early tracheostomy score (SETscore) was developed, and this tool has demonstrated moderate accuracy in predicting intensive care unit (ICU) length of stay (LoS), ventilation duration, and need for tracheostomy. We aim to assess the usefulness of SETscore in a more heterogeneous population that includes trauma patients to whom this score has not yet been applied.

Material and Methods: A retrospective consecutive analysis of all neurocritical patients who were admitted to our medical-surgical ICU between 2016 and 2018 and who required endotracheal intubation within 48 h of admission was performed in this study. Clinicodemographic data, as well as tracheostomy timing, imaging results, and SETscore were evaluated.

Results: The medical records of 732 neurocritical patients were reviewed, but only 493 patients were included, 68 of whom were tracheostomized (TR). These TR patients presented longer LoS and ventilation and antibiotic duration, lower Glasgow Coma Scale (GCS) score at admission, and more respiratory comorbidities. Severity scores, including SETscore, were higher in the TR group. A SETscore of >10 demonstrated 92.6% sensitivity and 79.1% specificity in predicting the need for tracheostomy. The majority of patients were tracheostomized after the seventh day of ICU admission. No significant differences in SETscore as well as in severity scores, age, and gender were observed between the early and late TR groups. However, the need for tracheostomy was significantly associated with lower ICU death rate even after controlling for GCS at admission, gender, age, and duration of invasive mechanical ventilation.

Conclusion: SETscore can be applied to a heterogeneous population. However, more data and prospective analyses are needed to validate their clinical usefulness on a daily basis. Nevertheless, the present data are expected to contribute to the management of neurocritical patients, particularly in the setting of ICUs managing a broad spectrum of critically ill patients.

## Introduction

Within the diverse setting of a medical-surgical intensive care unit (ICU), neurocritical care patients are a unique subgroup whose functional prognosis is extremely difficult to determine. As in most critical care patients, early weaning from a ventilator and extubation are desirable, as an extubation delay increases morbidity and mortality [1]. Tracheostomy is a routine procedure in polyvalent ICUs [2], and it is performed in 10% to 15% of patients. However, in neurocritical care patients, the reported rates are usually higher, ranging from 15% to 46.8% [3,4].

Not only is predicting successful extubation difficult, so is the decision to perform a tracheostomy. The lack of a standardized process in deciding for tracheostomy makes things even worse. Compared with a late tracheostomy, early tracheostomy seems beneficial in severe trauma neurocritical patients [5], who are generally ICU patients [6]; however, high-quality evidence to support this claim is lacking [2]. Apart from its being beneficial to patient comfort, tracheostomy reduces airway resistance, need for sedation and analgesia, and it may reduce the incidence of ventilator-associated pneumonia and other prolonged intubation complications, such as vocal cord injury and tracheomalacia [7-11].

Nonetheless, no standardized procedure is followed when deciding which patients will benefit from tracheostomy. The stroke-related early tracheostomy score (SETscore) was used in the pilot trial SETPOINT (stroke-related early tracheostomy versus prolonged orotracheal intubation in neurocritical care trial) and is related to the previously proposed TRACH (clinical and radiological predictors of tracheostomy in supratentorial spontaneous intracerebral hemorrhage) score [12,13]. In subsequent studies involving ventilated cerebrovascular neurocritical ICU patients [14,15], the SETscore demonstrated a moderate accuracy in predicting the ICU length of stay (LoS), ventilation duration, and tracheostomy need in Stroke neurocritical care patients.

We aim to assess the applicability of the SETscore and its usefulness in a more heterogeneous population consisting of ventilated neurocritical patients (including trauma patients) and ultimately to contribute to the standardization of the procedures involved in the decision-making process regarding tracheostomy.

## Materials and methods

We performed a hospital-based retrospective consecutive analysis of all neurocritical adult patients who were admitted to our polyvalent ICU between January 1, 2016 and December 31, 2018 and who required endotracheal intubation within 48 h of admission to the ICU.

The inclusion criteria were as follows: patients aged 18 and over; critically ill neurological patients admitted for neurosurgery; and patients with intracerebral hemorrhage (ICH), acute ischemic stroke (AIS), or subarachnoid hemorrhage (SAH) with or without traumatic cause and who required endotracheal intubation upon arrival to the emergency department or within 48 h of admission to the ICU. Patients who were intubated only for a scheduled procedure and extubated within 24 h were excluded. Demographic, clinical, and relevant outcomes were obtained from medical records and imaging results. All data were stored according to ethical principles and practices and according to data protection laws.

We calculated the SETscore of each patient as previously described [14]. Moreover, aiming to obtain homogeneous and comparable results, we took into account the definitions and clarifications of the score components proposed in the external validation study [15]. Similar to the previous studies, in this work, the data used to determine the SETscore was obtained from medical records, particularly the data collected within the first 48 h of admission and within 24 h of intubation.

Descriptive statistics was used to describe the baseline characteristics of the study population. Discrete variables were presented as absolute frequencies and percentages. Continuous variables, if normally distributed, were presented as mean ± standard deviation; otherwise, they are presented as median ± interquartile range.

A comparative analysis of tracheostomized (TR) and non-TR patients was performed in terms of demographic data (i.e., age and gender), clinical data (i.e., ventilation days, GCS, acute physiology and chronic health evaluation (APACHE) score, and ICU mortality rate), and all of the elements covered by the SETscore. These elements were analyzed as described in the most recent external validation study [15]. Early tracheostomy was defined as the tracheostomy performed within three to seven days from ICU admission. A SETscore of >10 was defined as a positive SETscore.

Variables were compared using the chi-square test or Fisher's exact test for categorical variables. Parametric and non-parametric data were compared using the Students’ t-test and the Mann-Whitney U test, respectively.

The receiver operating characteristics were obtained by calculating the area under the curve (AUC) with a 95% confidence interval (CI) to determine the performance of the SETscore in our study population. The Kaplan-Meier method was used to compare the survival between the patients with positive SETscore and the TR patients at one year post-ICU discharge. Cox proportional hazards regression analysis was used to estimate the implication of TR on one-year survival after adjusting for other prognostic factors such as age and comorbidities.

Data analysis was performed using the Statistical Package for the Social Sciences (SPSS®) 22.0 program (IBM, USA). Results were considered statistically significant at a two-tailed p-value <0.05.

## Results

The medical records of 732 neurocritical patients admitted to our ICU within a three-year period were reviewed. A total of 238 patients were excluded based on the exclusion criteria, leaving a final sample size of 493 patients, 68 of whom were tracheostomized and the 425 others were not.

The baseline characteristics of the study population are presented in Table 1. The two groups did not significantly differ in terms of age. The TR patients were more likely to be male, and this group presented longer LoS and ventilator and antibiotic duration as well as lower GCS score at admission. All calculated severity scores, including SETscore, were higher for the TR group. Moreover, the TR patients had a significantly greater number of respiratory comorbidities.

**Table 1:** Baseline population characteristics Baseline characteristics of the extubated and tracheostomized patients. Parametric/nonparametric variables are presented as mean ± standard deviation or median [interquartile range], respectively. Binary variables are presented as % (n). LoS: length of stay; GCS: Glasgow Coma Scale; APACHE: acute physiology and chronic health evaluation; SAPS: simplified acute physiology score; ICU: intensive care unit

	Extubated (n=425)	Tracheostomized (n=68)	p Value
Age (years)	58.66 ± 15.87	58.57 ± 16.23	0.967
Male	47.20 (201)	66.18 (45)	<0.001
LoS (days)	3.89 ± 4.89	18.24 ± 10.60	<0.001
GCS at admission	11.90 ± 4.09	6.75 ± 3.58	<0.001
Ventilator days	3.12 [3.0-16.0]	18 [11.0-24.0]	<0.001
Antibiotic days	3.76 ± 3.69	12.27 ± 7.44	<0.001
APACHE > 20	23.76 (101)	66.18 (45)	<0.001
SAPS II	31.04 ± 19.72	49.43 ± 14.44	<0.001
SAPS III	36.08 ± 16.66	50.62 ± 12.96	<0.001
Respiratory comorbidity	11.53 (49)	19.12 (13)	<0.001
SETscore	6.27 ± 5.47	16.99 ± 4.68	<0.001
ICU mortality	13.20 (56)	1.47 (1)	<0.001
1-year mortality	117 (31.7)	30 (44.8)	0.009

The frequency of certain diagnosis at admission and the tracheostomy risk of the two groups are compared in Table 2. Traumatic lesions (e.g., SAH and others) were associated with a higher odds ratio (OR) for tracheostomy (5.108 and 4.923, respectively, both p < 0.001). Meanwhile, it is noteworthy that only one patient with a CNS tumor was submitted to tracheostomy.

 

**Table 2:** Diagnostics at admission Different diagnosis and association with the need for tracheostomy. Binary variables are presented as % (n). Odds ratio versus tracheostomy risk in all other diagnosis is presented as n ± CI95%. AIS: acute ischemic stroke; ICH: intracerebral hemorrhage; SAH: subarachnoid hemorrhage; SDH: subdural hematoma; CNS: central nervous system

	Extubated	Tracheostomized	Odds ratio	p Value
Diagnosis at admission (n)	-	-		<0.001
AIS (31)	83.87 (26)	16.13 (5)	1.218 (0.451-3.289)	0.6
ICH (51)	76.47 (39)	23.53 (12)	2.121 (1.048-4.293)	0.033
Traumatic SAH (19)	57.89 (11)	42.11 (8)	5.018 (1.941-12.977)	0.002
Non traumatic SAH (69)	82.61 (57)	17.39 (12)	1.383 (0.699-2.739)	0.350
SDH (46)	78.26 (36)	21.74 (10)	1.863 (0.877-3.956)	0.101
Other traumatic lesions (41)	60.98 (25)	39.02 (16)	4.923 (2.467-9.823)	<0.001
CNS tumor (198)	99.49 (197)	0.51 (1)	0.017 (0.02-0.126)	<0.001
Other diagnoses (38)	89.47 (34)	10.53 (4)	0.719 (0.247-2.094)	0.543

**Figure 1:**
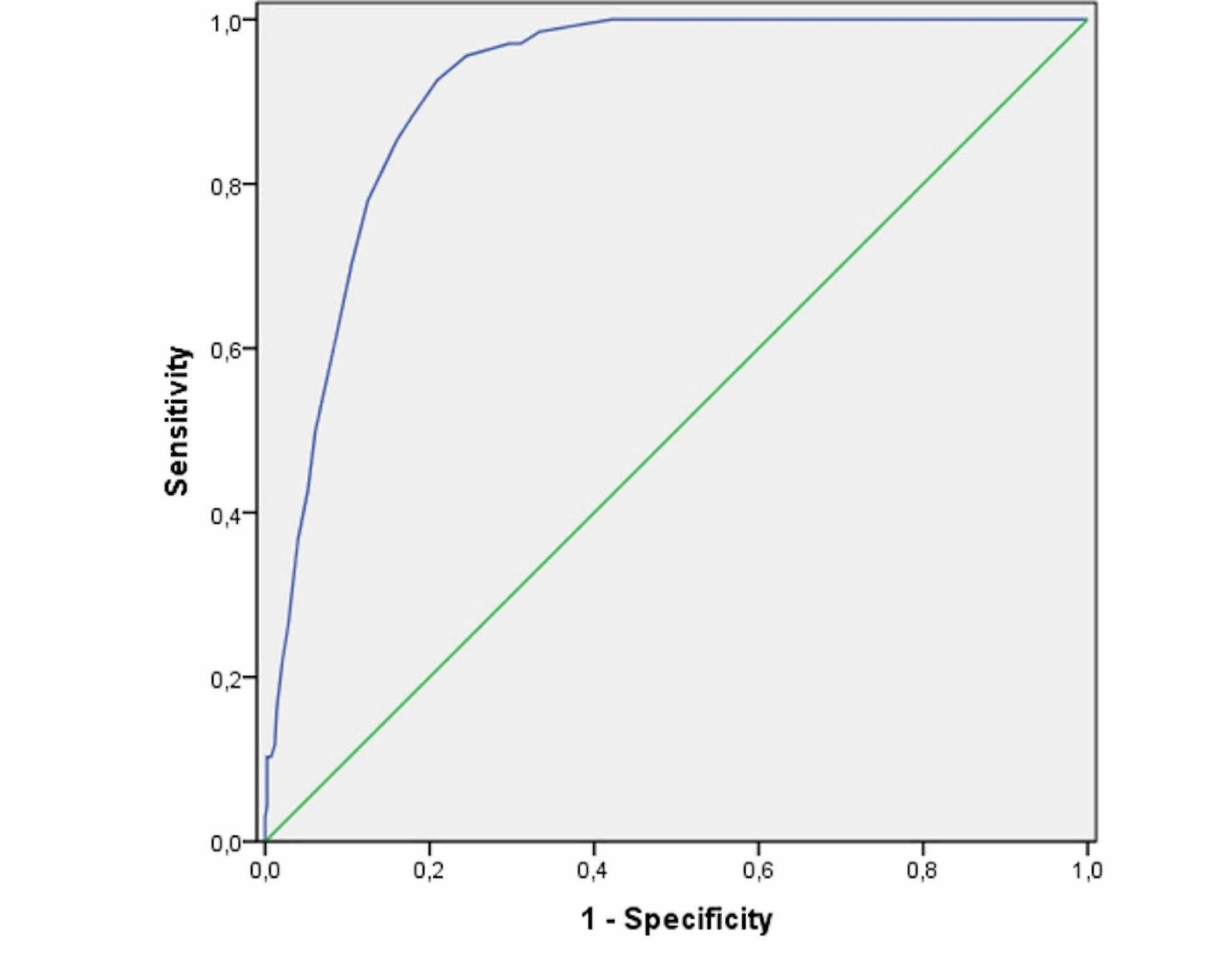
Area under the receiving operating characteristics for discrimination of tracheostomized and non-tracheostomized patients by SETscore. SETscore: stroke-related early tracheostomy score

Regarding the ability to distinguish between TR and non-TR patients based on the SETscore, when a cut-off value of >10 points was used to predict the need for TR (as proposed in previous studies), the sensitivity was 92.6% (95%CI, 83.0-97.3) and the specificity was 79.1% (95%CI, 74.8-82.8). The accuracy was 80.9% (95%CI, 77.2-84.3) with an AUC of 0.91 (95%CI 0.89-0.94) (Figure 1). A higher cut-off value of 15 points would increase the specificity to 91.3% (95%CI, 88.1-93.7) but at the expense of a considerable reduction in sensitivity to 61.8% (95%CI, 49.1-73.0).

 

The need for tracheostomy was significantly associated with a lower ICU death rate (p < 0.01; hazard ratio (HR) 10.16; 1.38-74.71). Only one TR patient died during the ICU stay. Moreover, tracheostomy timing (early vs late) was not associated with a higher ICU mortality rate (p = 1.0). In the TR patients, neither ICU LoS (p = 0.08) nor antibiotic duration (p = 0.946) were associated with higher ICU mortality. A low CGS score at admission was significantly associated with higher ICU mortality (6 vs 14; p < 0.01). High severity scores (simplified acute physiology score (SAPS) II, SAPS III, and APACHE) at admission were associated with higher mortality. Out of the 57 patients who died during their ICU stay, 46 had a SETscore of >10. However, 44 of those were already admitted with a very poor prognosis or with the intent to become organ donors; therefore, they were not considered for tracheostomy (non-TR group).

**Figure 2:**
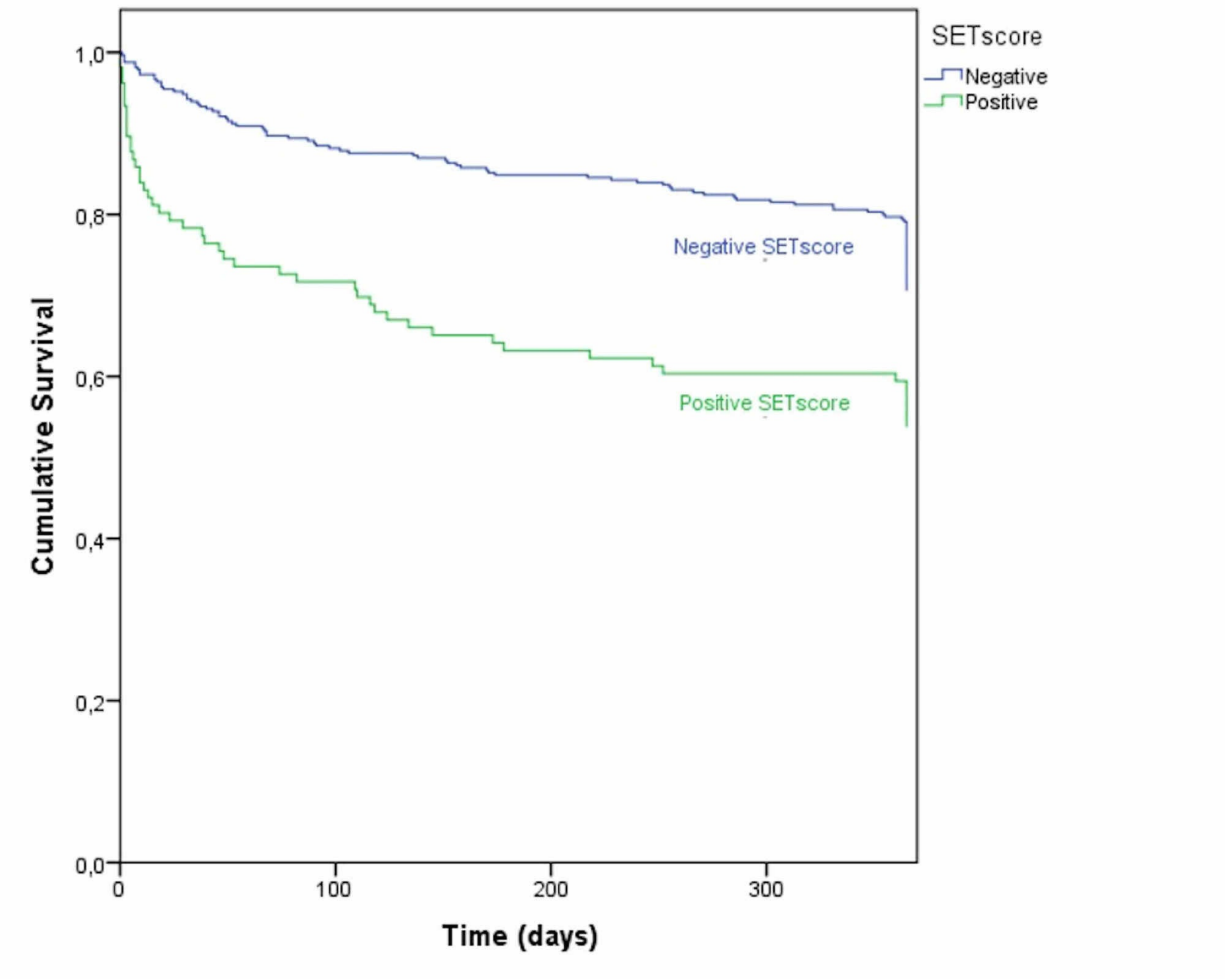
Kaplan-Meier curve of one-year mortality according to SETscore result. SETscore >10 considered positive. SETscore: stroke-related early tracheostomy score

Several variables were associated with one-year mortality after ICU discharge; these variables were positive SETscore (OR 2.035; 95%CI, 1.299-3.188; p = 0.002), male gender (OR 1.930; 95%CI, 1.290-8.887; p = 0.001), older age (p = 0.001), longer duration of invasive mechanical ventilation (p = 0.001), and lower GCS score at admission (p = 0.032). The need for tracheostomy was also significantly associated with one-year mortality (OR 1.746; 95%CI, 1.029-2.964; p = 0.037), but there were no differences regarding tracheostomy timing (OR 1.517; 95%CI, 0.399-5.769; p = 0.742). Using the Kaplan-Meier statistics (Figure 2), we found an association between a positive SETscore and a lower one-year survival post-discharge.

**Figure 3:**
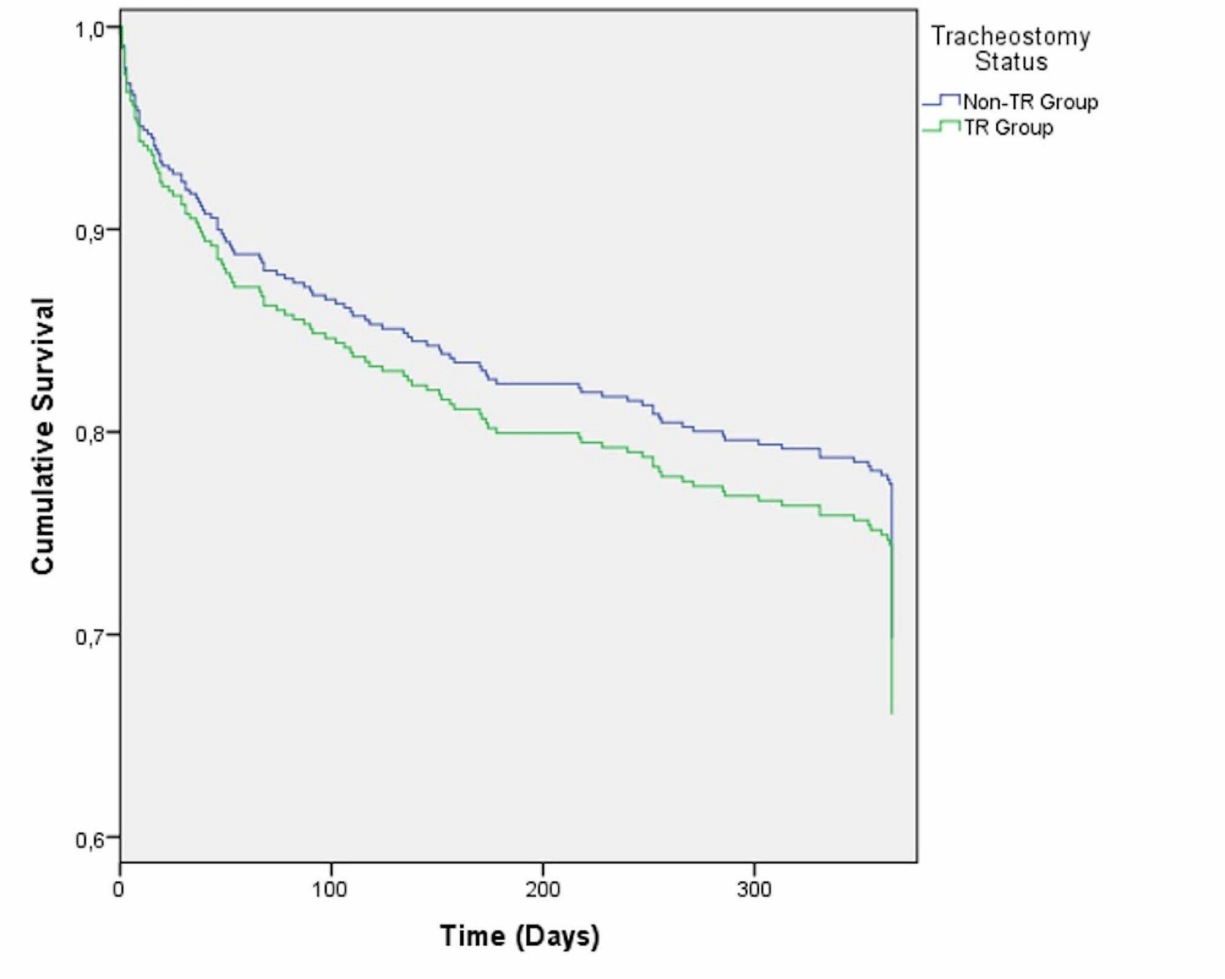
Cox regression proportional hazards for one-year mortality according to tracheostomy status. TR group: tracheostomized patients; non-TR group: non-tracheostomized patients

The multivariate analysis controlling for age, gender, length of invasive mechanical ventilation, and GCS score at admission showed no significant association between TR and one-year survival post-ICU discharge (HR 0.886; 95%CI, 0.525-1.430; p = 0.574) (Figure 3).

## Discussion

Tracheostomy in neurocritical patients remains a subject of debate given that the timing and the selection of patients who might benefit from this strategy remain unknown [16].

Our study evaluated the external validity of a tool that aids in the selection of patients who will benefit from tracheostomy. Usually, GCS, APACHE II, SAPS II, and Abbreviated Injury Scale are used to define illness severity and are transposed as decision-making tools regarding the need for tracheostomy in neurocritical patients [17,18].

In an attempt to standardize procedures, we included patients with traumatic brain injury as well and applied the SETscore to all patients. To our knowledge, this study is the first to include and apply the SETscore to these patients. This score was initially developed as a screening tool based on tracheostomy predictors identified in several retrospective studies, that were combined under three different categories: neurological function, neurological lesion, and general organ function [14]. We applied the SETscore to all the neurocritical patients admitted within the described period.

Even when used in the more heterogeneous population in this study, the SETscore has proven to be highly discriminative to predict the need for tracheostomy, with an AUC of 0.91. The obtained results are consistent with a reported finding [15]. Sensitivity and specificity were better for the previously proposed cut-off value of 10 points (92.6% and 79.1%, respectively). The specificity could have been higher had the patients with poor short-term prognosis at admission been excluded (i.e., the 44 patients who were not candidates for tracheostomy despite their SETscores). As such, one can expect that the performance of the SETscore is slightly better in the real-world setting.

It is important to highlight that among the TR patients, nine were subjected to the procedure after at least 20 days in the ICU. Therefore, in patients with similar severity scores, the SETscore could assist in the selection of patients who will need tracheostomy; thus, the SETscore demonstrates a predictable positive impact not only to the patient but also to the healthcare system.

A positive SETscore and the need for TR were associated with higher one-year mortality, possibly indicating the effect of a more serious neurological injury at admission. Longer weaning times have been associated with worse post-ICU outcomes [19]. Nonetheless, when possible confounding factors, such as length of invasive mechanical ventilation, were controlled, no significant differences between the TR and non-TR groups were noted. This finding reinforces the notion, as stated in the literature [12,20], that tracheostomy is a feasible procedure in selected cases with short-term benefits (reduced ventilation duration and ICU stay) and without deleterious effects at longer follow-up periods.

Despite the limitations of this study (single-center retrospective analysis of inherently limited population and data; SETscore was evaluated retrospectively), the data obtained and the results achieved are consistent with previous findings, clearly demonstrating the high sensitivity and specificity of SETscore. Given the previously reported accuracy of the SETscore and given that it has just been evaluated in a population that includes other type of patients in a polyvalent ICU, the SETscore is a potentially good tool to help decide if and when tracheostomy is beneficial.

Further studies are warranted to understand the neurological outcomes of the ICU survivors.

## Conclusions

This analysis shows that SETscore can be applied and can provide useful information about our study population, which included both trauma and non-trauma neurocritical care patients. More data is needed, and a prospective analysis will be beneficial and may conveniently prove the usefulness of SETscore on a daily clinical basis. Furthermore, this score may be particularly helpful in Units similar to ours, where neurocritical patients represent a small fraction of patients. It can guide us through the decision for early tracheostomy with the inherent benefits.

The present data is expected to contribute to changing the approach and management of neurocritical patients not only in our ICU but also in other polyvalent ICUs having similar characteristics and facing the same difficulties.
